# SOCIAL DETERMINANTS AND THE VULNERABILITY TO LOSS OF INDEPENDENCE IN OLDER ADULTS

**DOI:** 10.1093/geroni/igae098.2815

**Published:** 2024-12-31

**Authors:** Young-Shin Lee, Hee-jin Jun, Jung-Ah Lee

**Affiliations:** San Diego State University, San Diego, California, United States; San Diego State University, San Diego, California, United States; University of California Irvine, Irvine, California, United States

## Abstract

Over 41% of Americans with disabilities are aged 65 and older, with two in five seniors experiencing at least one disability, such as mobility limitations or depending on others for daily activities. As life expectancy increases, the prevalence of disability in later life is also expected to increase. Vulnerability of independence and unmet needs in daily activities present a significant personal burden and a growing concern for public health. While mMobility disabilities contribute significantly to this vulnerability, yet a comprehensive understanding is still lacking. This study aims to explore the impact of social determinants on functional vulnerability of older adults, utilizing data from the 2017 National Health Aging Trends Study (NHATS). It evaluates functional vulnerability with the Vulnerable Elders Survey tool and applies logistic regression analysis to assess the effects of age, gender, race/ethnicity, income, and living arrangements on this vulnerability. The analysis included 6,312 individuals aged 65 years and above, revealing that 30% have an income under the poverty level and 35% live alone. The results show that 43.6% of older adults are at a high risk of functional vulnerability. Significant risk factors identified include being of ‘old’ age, female, NH Black, living with others, and having an income at the poverty level. These findings underscore the urgency of developing targeted interventions for these at-risk groups.

